# Opportunities for Increasing Resilience and Sustainability of Urban Social–Ecological Systems: Insights from the URBES and the Cities and Biodiversity Outlook Projects

**DOI:** 10.1007/s13280-014-0505-z

**Published:** 2014-04-17

**Authors:** Maria Schewenius, Timon McPhearson, Thomas Elmqvist

**Affiliations:** 1 Stockholm Resilience Centre, Stockholm University, Kräftriket 2B, 106 91 Stockholm, Sweden; 2 Tishman Environment and Design Center, 79 Fifth Avenue, 16th Fl., The New School, New York, NY 10003 USA

**Keywords:** Urbanization, Ecosystem services, Governance, URBES, CBO

## Abstract

Urban futures that are more resilient and sustainable require an integrated social–ecological system approach to urban policymaking, planning, management, and governance. In this article, we introduce the Urban Biodiversity and Ecosystem Services (URBES) and the Cities and Biodiversity Outlook (CBO) Projects as new social–ecological contributions to research and practice on emerging urban resilience and ecosystem services. We provide an overview of the projects and present global urbanization trends and their effects on ecosystems and biodiversity, as a context for new knowledge generated in the URBES case-study cities, including Berlin, New York, Rotterdam, Barcelona, and Stockholm. The cities represent contrasting urbanization trends and examples of emerging science–policy linkages for improving urban landscapes for human health and well-being. In addition, we highlight 10 key messages of the global CBO assessment as a knowledge platform for urban leaders to incorporate state-of-the-art science on URBES into decision-making for sustainable and resilient urban development.

## Introduction

Urbanization is an important driver of land-use and land-cover change (Eigenbrod et al. 2011; Elmqvist et al. 2013), which in turn alters biodiversity and the delivery of critical ecosystem services of importance for urban resident health and well-being (Seto et al. 2013). The pressure is increasing on urban planners and policymakers to direct urban growth and development toward increased protection of ecosystems both within and outside cities that produce many critical resources used in the cities.

The observed global erosion of the ability of ecosystems to generate services (MA 2005) not only demands increased understanding of the relationship between urban biodiversity and human health and well-being, but also requires that this knowledge be quickly translated into urban planning, management, policymaking, and governance (Carpenter et al. 2009; TEEB 2010).

Urbanization patterns are still unclear with respect to future locations, magnitudes, and rates of urban expansion (Seto et al. 2012; Fragkias et al. 2013), predictions of how urbanization affect the functioning of urban and peri-urban ecosystems, and therefore the generation of ecosystem services remains limited (Kremen and Ostfeld 2005; Elmqvist et al. 2010, 2013). How urban development can best be designed to support the provisioning of ecosystem services needs much additional research (Butler and Oluoch-Kosura 2006; Elmqvist et al. 2010; Marcotullio and Solecki 2013).

Underpinned by the global framework provided in the CBO, this article focuses on the development trends of five of the case-study cities of the URBES project: Barcelona, Berlin, New York, Rotterdam, and Stockholm. Here, we contextualize the findings from the URBES project and provide insights on the relationships between urban development trajectories, urban ecosystems, urban planning and management, and human well-being. We discuss key findings in the URBES and CBO projects, and how they can be used to increase the capacity of urban planning and management to utilize urban ecosystems for human health and wellbeing. The primary question guiding this article is: What are the key findings in the URBES and CBO projects that can be used to increase the capacity of urban planning and management to utilize urban ecosystems for human health and wellbeing?

## The Importance of a Social–Ecological Context to Understand Urban Resilience and Sustainability

It has been argued that resilience^1^ is a necessary approach to meet the challenges of sustainable development^2^ (Chelleri and Olazabal 2012; Elmqvist et al. 2013). Cities that are designed and developed using sustainability and resilience best practices, can support or even enhance the capacities of ecosystems in and around cities to provide services (ICLEI 2013). For example, cities can themselves increasingly be important partial sources of energy, food, and fresh water production, as well as homes for rich biodiversity (Gómez-Baggethun et al. 2013). In order to build local resilience that also supports desired resilience elsewhere, it is important that solutions for urban regions take into account the far-stretched impacts on, and connections with, the rest of the planet (Elmqvist et al. 2013).

The social and ecological context of urban geographies influences resilience to urbanization, climate, and other social–ecological challenges (Marcotullio and Solecki 2013). For example, the biogeophysical context of the city or urban area may determine how ecosystems respond to rapid urbanization, climate change and extreme events (Solecki et al. 2011). In addition, social, institutional, economic, and political contexts will also influence ecological resilience. As social–ecological systems, cities may be small, large, or mega; coastal, desert, mountainous, tropical, or island. They may have similar system characteristics and even similar development and ecosystem impact trajectories (Romero-Lankao and Dodman 2011). More often than not, however, they differ, with some expanding, while others are shrinking; with some building up, while others build out; and with some experiencing several trends running in parallel in the same city. In addition, some cities sprawl, even while their populations simultaneously decline (Schmidt 2011).

Still, we know that common impacts of urban growth include increases in sealed surfaces, urban sprawl, traffic congestion, and residential segregation (PLUREL 2011). In contrast, shrinking cities often result in vacant urban land areas (Haase et al. 2012; Kremer et al. 2013; McPhearson et al. 2013b). It will, therefore, be important to build up local case studies of best practices in multiple urban contexts to better understand the general versus locally driven relationships between urbanization, ecosystem functioning and service provisioning, and more, how to operationalize successful strategies in cities worldwide.

Since expected urban land-use changes differ from city to city, often with country-specific drivers (Siedentop and Fina 2012), we suggest that it will be difficult to prescribe one-size-fits-all solutions for urban sustainability (Elmqvist et al. 2013) and resilience. Local governance adapted to local challenges and conditions will be important. Still, some general rules for improving sustainability and resilience in cities and urbanized regions are emerging, as evidenced by the Cities and Biodiversity Outlook (CBO) assessment and Urban Biodiversity and Ecosystem Services (URBES) research (see this special issue).

## Urbanization Trends in Europe and North America

North America contains some of the most urbanized landscapes in the world. In the United States (US) and Canada, approximately 80 % of the population is urban (UN-DESA 2011). Between 1970 and 2000, urban land area in North America expanded at a rate of 3.31 % (Seto et al. 2011), creating unique challenges for conserving biodiversity and maintaining regional and local ecosystem services (McPhearson et al. 2013a).

Although both Europe and North America have historical urbanization patterns of growth and sprawl (Marcotullio and Solecki 2013), more recent urbanization differs between the different regions, between cities within these regions, and indeed within cities and their hinterlands. Urban land area in Europe and North America is expected to increase or remain constant by 2030 compared with 2000 (Kabisch and Haase 2011; Seto et al. 2012). These calculations, however, only include new land areas and mask intra-urban land change dynamics including intensification and densification.

Urbanization patterns within urban regions and cities are more nuanced. In Europe, for example, medium- and large-sized cities continue to expand, while smaller cities are often in decline (Kabisch and Haase 2011). Although urban populations in many European and North American cities are still increasing, overall rates are slowing (UN-DESA 2011). While many cities in the U.S. experience population decline, the population of megacities such as New York are growing (McPhearson et al. 2013a). The correlation between population and land area trends is not necessarily clear either; for example, while the population of the city of Berlin has stabilized, city boundaries continue to expand (Lauf et al. 2012).

Many urban challenges today are global in scope (e.g., climate-change effects such as sea level rise). Geographically distant and socioeconomically differing cities, for example, Rotterdam and Jakarta, will still need to prepare for similar challenges (Ward et al. 2013). The insights from the URBES case-study cities in Europe and the US, and the CBO Scientific Foundation, with its comprehensive global analyses, can thus be immediately relevant to support the development of guidelines for governance of urban ecosystems around the world.

### URBANIZATION IN THE MOST RAPIDLY GROWING AND DEVELOPING REGIONS

Although with large regional disparities, the overarching urbanization patterns in Asia, Africa and Latin America differ dramatically from the current patterns present in Europe and the US. While the general trends are of rapidly growing urban populations and urban sprawl, some cities, for example, the megacity of Tokyo, experience a significant decline or even reversed growth rate (Kohsaka et al. 2013), following patterns otherwise mostly seen in the US and Europe.

**Fig. 1:**
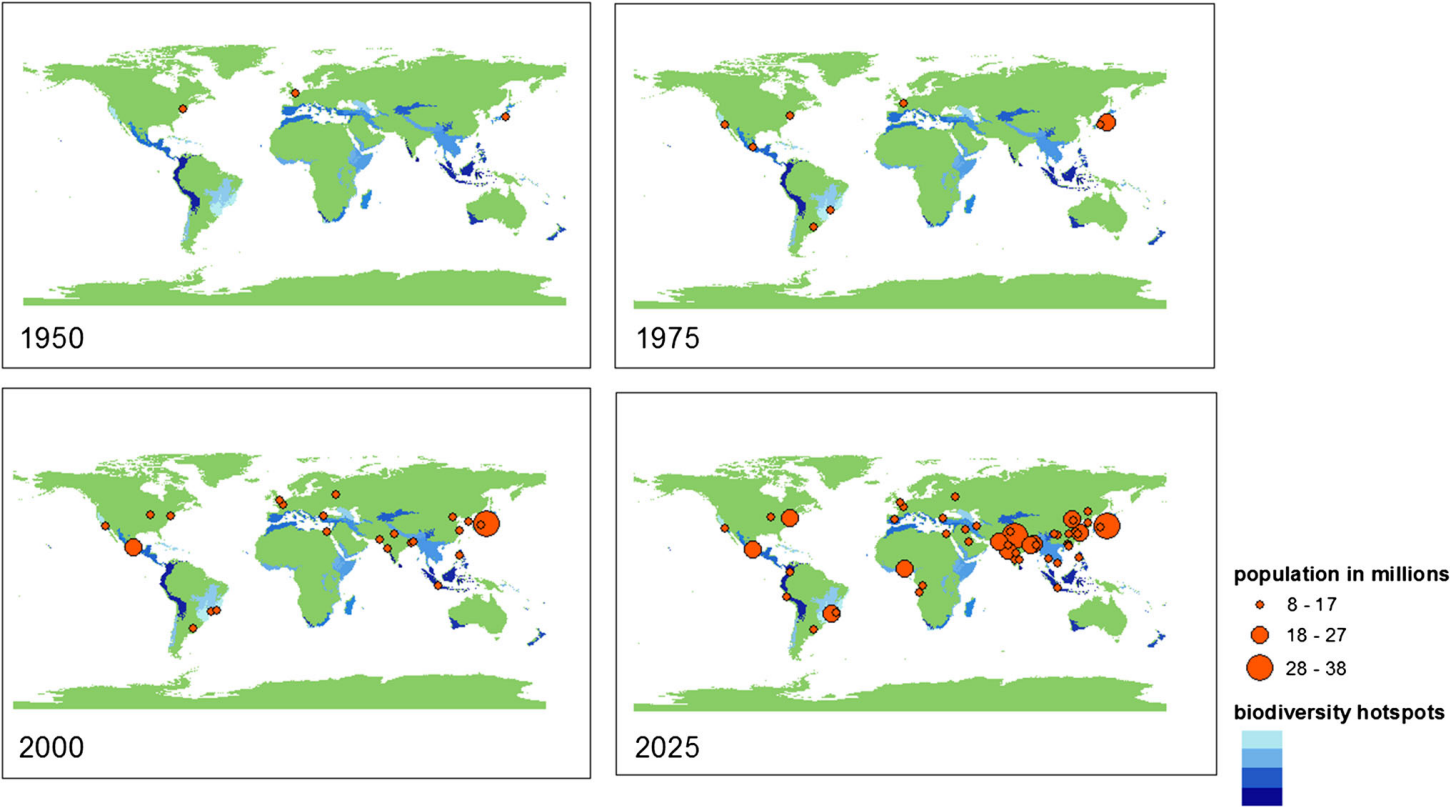
Urban areas (in *orange*) with large populations in 1950, 1975, 2000, and 2025 (projected), as examples of urban expansion in global biodiversity hotspots, shown with higher (*dark blue*) and lower (*light blue*) levels of biodiversity (Image credit: Femke Reitsma, University of Canterbury)

While the regions of Africa, Asia, and Latin America experience the most rapid urban growth and development (Fig. 1), they are simultaneously under-represented in international scientific literature on the topic (Wang et al. 2012; Wilkinson et al. 2013).

The high population growth and rural-to-urban migration makes Africa the fastest urbanizing continent, with a 3.4 % urban population increase annually (Anderson et al. 2013). Acknowledging that large regional differences within the continent exists, urban populations at large are expected to increase significantly, as still only 40 % of the continent’s total is currently urban (Anderson et al. 2013).

Similarly, many parts of Asia are experiencing rapid development and growth, with large regional differences. Many countries that are predominantly rural, including Bangladesh, Vietnam, and India, are undergoing massive demographic transitions resulting in rapidly growing urban populations (Seto 2013). Half the increase in urban land globally over the next 20 years will occur in Asia, with the most extensive patterns of change expected to take place in India and China (Seto 2013).

In Latin America, sprawl rather than population growth is expected to be the predominant challenge in the future. Similar to the US and Canada, more than 80 % of the population in Latin America lives in cities, and this is expected to increase to 90 % by 2050 (Pauchard and Barbosa 2013). Industrialization of agriculture has caused abandonment of poor soils, but land-use changes today are increasingly the result of urbanization. Latin American cities have on average grown sixfold over the past 50 years, resulting in densely populated cities and increasingly abandoned rural areas (Pauchard and Barbosa 2013).

Globally, urban growth is not only unprecedented in the rate and sheer number of urban residents, but it is also quickly expanding into the world’s most biodiversity rich areas (Fig. 1) (Seto et al. 2012; Güneralp et al. 2013). This expansion into the world’s remaining hotspots for species and genetic diversity has implications for both urban and global biodiversities, and by extension, for both urban and global ecosystem services provisioning. The knock-on effects of land-use changes outside of cities, which, for example, can include damming of rivers, water diversions, and agricultural practices (Seto 2013), can also have effects on the capacities of ecosystems inside cities to function and produce services (Ignatieva et al. 2010).

## URBES

### The URBES Project

The URBES project (www.urbesproject.org) recognizes and aims to bridge knowledge gaps on the role of URBES for human well-being, and the need for the increased capacity of cities to adapt to climate change. As a collaborative research project among 11 top research centers in Europe and North America, URBES is developing guidelines for integrating ecosystems in urban landscapes and for monetary and nonmonetary valuation of ecosystem services. The project actively links to important policy mechanisms and contributes to global partnerships, such as the Convention on Biological Diversity (CBD), The Economics of Ecosystems and Biodiversity (TEEB), the Intergovernmental Platform on Biodiversity and Ecosystem Services (IPBES) (Box 1), and the European Union on the post-2010 EU Biodiversity Strategy. URBES is organized into nine research work packages, which focus on defining urban biodiversity within and around cities, assessing and developing monetary and nonmonetary valuation evaluation models, understanding current governance and management of URBES, and actively engaging with urban decision-makers, planners, and practitioners through dialogues and knowledge exchange.

**Box 1:** Research and policy on a global scale

In addition to the URBES Project and the CBO, two major new global networks have been established to increase knowledge exchange between policy-makers and scientists. IPBES, the Intergovernmental Platform on Biodiversity and Ecosystem Services, was established in 2012 (IPBES 2013) to provide policy-relevant knowledge on biodiversity and ecosystem services, and increase effectiveness in conserving biodiversity and ecosystem services (Pe’er et al. 2013). IPBES is intended to be able to quickly develop new research themes on emerging issues, and respond to questions from both governmental and nongovernmental organizations, as well as the public (Pe’er et al. 2013)
Also in 2012, a new platform, Future Earth, merged the four global change research programmes *International Geosphere*—*Biosphere Programme, International Human Dimensions Programme*, *Diversitas*—*An International Programme of Biodiversity Science*, *and World Climate Research Programme and Earth Science System Partnership*, as a response to the urgent call for “an ethical framework for global stewardship and strategies for Earth System management” (Biermann 2007). The establishment of Future Earth marks a transition to a global institutional framework with a focus on *earth systems*. The new platform is intended to be responsive to the changing needs and priorities of decision-makers at regional and national level, and disseminate knowledge and capacity on science for sustainability across the globe (ICSU 2013)

In addition to peer-reviewed publications (see articles in this special issue), short research summary guidelines for policymakers and planners have been produced throughout the project, to communicate findings and provide governance support. A video has also been produced, and more such guidelines are planned, to present the project to the public and highlight ongoing best practices. Finally, the project hosts workshops and trainings with representatives from cities around Europe not only to directly contribute to training for professionals but also to provide mechanisms for stakeholder knowledge to contribute to project outcomes.

### Insights from the URBES Case-Study Cities

#### Novel Plans and Guidelines for the Future of Barcelona

The Barcelona Metropolitan Region, where the city of Barcelona, Spain, is located, is one of the most densely populated regions in Europe, with a land area of 3200 km^2^ and a population of 4.5 million (Marull et al. 2010). Supported by a near absence of ecological considerations in urban plans (Paül and Tonts 2005), the city of Barcelona has during the last five decades expanded at a considerable rate, and roughly 1000 ha/year of rural land has been converted to urban uses (Marulli and Mallarach 2005).

The Barcelona Green Infrastructure and Biodiversity Plan, which was released in 2013, is a novel approach in the planning of the city, falling in line with the *EU Biodiversity Strategy to 2020* (European Commission 2011) and the Aichi targets for 2011–2020 (CBD 2010). Acknowledging that “it is vital to strive towards a city where nature and urbanity converge and enhance one another” (Ajuntament de Barcelona 2013; see also Baró et al. 2014; Langemeyer et al. 2014), one of the plan’s primary aims is to increase connectivity among patchy green infrastructure. However, Barcelona’s largest challenge may not be the green spaces inside the city, but rather land-use changes in the surrounding areas. If the urban sprawl of the past few decades continue (Muñiz et al. 2013), then the region’s biodiversity and basic ecological processes, which are dependent on the traditional mosaic of land practices and network of protected areas, may be significantly negatively impacted (Marull et al. 2010). Thus, focusing green infrastructure planning on increasing connectivity among existing green spaces will be important for safeguarding urban ecosystem services in a region characterized by sprawl.

#### Overlapping Protection Models by Formal and Informal Management Systems in Stockholm

The role of informal management as an addition to formal guidelines has been explored in another URBES case-study city, Stockholm. The Stockholm County constitutes Sweden’s most populated and rapidly growing region, currently with about 2 million inhabitants (Statistics Sweden 2011). As the population increases and the city grows, mainly through densification, the green areas are decreasing in numbers and sizes (Colding 2013). Ecosystem management in the Stockholm region is conducted along municipal governance lines, but the system of self-governing local municipalities challenges the goals of regional sustainable development (Colding 2013). At the same time, it has been argued that the sustainability of the urban landscapes is dependent on reducing or eliminating the disconnection in management on different scales, whether operational, tactical, or strategic (Borgström et al. 2006). The contribution to green area management from informal management is substantial and important, for example, as management of allotment gardens, golf courses, and domestic gardens (Ernstson et al. 2010; Andersson et al. 2014). Collaborative ecosystem management in Stockholm has been found to actively include not only new knowledge but also a wider set of values through interaction in social networks (Ernstson et al. 2008). Creating strong links between informal managers and formal governance can thus be crucial to form holistic governance and successful management outcomes. However, informal management is today seldom translated into informal governance in urban settings, which could lead to local self-organization around ecosystem management being hampered (Colding 2013).

#### Civic Engagement for Urban Green Space Management in Berlin

Berlin, Germany, is with a population of 3.5 million (Senatsverwaltung für Stadtentwicklung und Umwelt 2012), one of the most populated and simultaneously one of the greenest cities in Europe. Informal management of the city’s green spaces is an example of how management can support resilience in cities. Civic engagement in green space management has been encouraged by local politicians as funding for public parks has decreased (Rosol 2010). Different types of commonly managed urban greens can be found in Berlin, for example, the Burgerparks, representing public parks managed by 10–100 local residents each, and Public-Access Community gardens, open for anyone at all times and managed by various interest groups (Bendt et al. 2013). Management by locals holds promise to foster learning and maintain social–ecological memories connected to a place or an activity, which in turn can support biodiversity conservation (Barthel et al. 2010; Bendt et al. 2013). Furthermore, the coalitions of managers have been found to promote cultural integration, and increase the capacity of cities to deal with sudden changes, such as unemployment and economic recessions (Colding and Barthel 2013). A recent study of importance for urban green space planning, however, indicates that access to green spaces in the city is unequally distributed among different demographic groups, and that the qualities of the green spaces do not always match the needs of the different user groups (Kabisch and Haase 2014).

#### Green Infrastructure Solutions in the Metropolis of New York

New York City (NYC) has a population of over 20 million in the metropolitan region, with over 8 million within the city’s municipal boundaries. The city contains the most parkland of any US city, and has 21 % covered by tree canopy, which is expected to increase over the coming decades. NYC is an example of how cities can harbor rich biodiversity, with 85 % of the floral diversity of New York State existing within the city (McPhearson et al. 2013c).

Still, the local biodiversity of NYC is faced with significant challenges, including pollution, climate change, sea level rise, stormwater management, and human population growth (McPhearson et al. 2014). Urban planning, management, and policy-making have begun to invest in green infrastructure (NYC 2010) as a cost-effective tool for achieving sustainability and resilience goals in the city (McPhearson 2011). The sustainable infrastructure investment approach addresses multiple goals using “blue roofs,” larger street tree pits, “green streets,” porous concrete, and vacant lots to control stormwater and provide additional ecosystem services (Cohen and Ackerman 2011; McPhearson et al. 2013c). The NYC Green Infrastructure Plan (NYC 2010) commits a total of US$2.4 × 10^9^ over 20 years to control 10 % of stormwater runoff using green infrastructure. It is estimated that the socially and ecologically damaging combined sewage overflows into nearby rivers, streams, and wetlands will be reduced by approximately 1.5 × 10^9^ gallons per year (NYC 2010). This plan showcases the importance of urban green space for local ecosystem services benefits to the city.

#### Climate Change Effects in Low-Lying Coastal City of Rotterdam

The city of Rotterdam is a low-lying delta city population of which has remained fairly stable at around 1 million residents for the last decade. Its geographic position makes the city and port increasingly vulnerable to climate change effects, such as flooding due to sea level rise, rivers with increasing peak discharges, and changing precipitation patterns (Meyer et al. 2012; Ward et al. 2013). A shift is taking place in urban management, moving the focus from technological flood mitigation strategies to risk-based and adaptation strategies (Ward et al. 2013). It has been argued that increasing the city’s capacity to meet the growing challenges is to a large extent dependent on developing a holistic governance approach where the city is understood as a dynamically interacting social–ecological system (Frantzeskaki and Tilie 2014). Increased linkages between strategies, projects, and actors (Meyer et al. 2012), including the active involvement of local citizens, have been identified as a key factor to identify needs and challenges, design policies, and efficiently implement them (Wardekker et al. 2010; Ward et al. 2013).

### Summary

The five case studies discussed above highlight sustainability trends, ongoing challenges, and novel opportunities in four European and one North American city. However, a deeper understanding is needed of the diverse and often overlapping urbanization trends in cities across the world, and the capacities to deal with expected climate changes and the impacts on biodiversity. One significant output of the URBES project has aimed to increase this understanding and guide adaptation of solutions to local conditions.

## Cities and Biodiversity Outlook: A Global Assessment

**Fig. 2:**
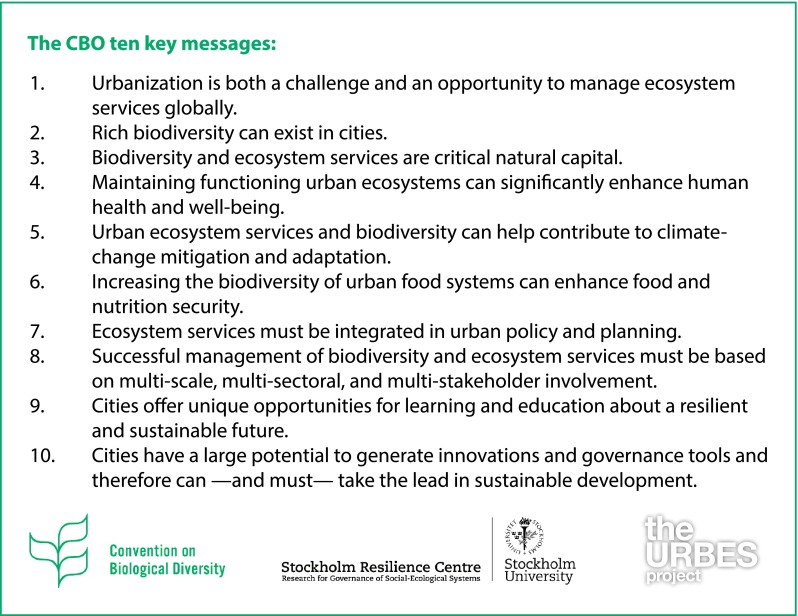
The 10 key messages as detailed in CBO—*Action and Policy*. *Source* www.cbobook.org/resources

The Cities and Biodiversity Outlook, a project linked to URBES and springing from the U.N. Convention on Biological Diversity, recently published its Scientific Foundation: *Urbanization, Biodiversity and Ecosystem Services*: *Challenges and Opportunities. A Global Assessment* (Elmqvist et al. 2013). The book is the world’s first global assessment on global urbanization trends, with their associated links to, and impacts on, ecosystems. The book follows a previously published report from the project, CBO—*Action and Policy* (Secretariat of the Convention on Biological Diversity 2012), which provides insights and guidelines for decision-makers on urban development and planning in support of ecosystems. CBO— *Action and Policy* presented 10 key messages (Fig. 2) aimed at not only generating awareness but also illustrating important steps on a pathway toward urban sustainability and resilience.

URBES research partners played a significant role in developing the CBO Scientific Foundation (Elmqvist et al. 2013). The world’s first assessment of its kind, the Foundation provides a new basis for future understanding of the relationship between urbanization patterns, land-use change processes, governance mechanisms, and urban policy, planning, and management with the goal of achieving livable cities with healthy ecosystems and residents.

The CBO project aimed not only to synthesize and create new knowledge, but it also actively worked, like the URBES project, to reach beyond the academic community to communicate its findings to decision-makers, planners, practitioners, and the general public. Outputs from the project thus include, in addition to the report *Action and Policy*, the book *Urbanization, Biodiversity and Ecosystem Services*: *Challenges and Opportunities. A Global Assessment*, the short video *An Urbanizing Planet* and the website www.cbobook.org, where the material produced throughout the project is freely downloadable, together making the information accessible for a wide audience.

## Discussion

### Remaining Governance Challenges

Despite new research initiatives and science–policy platforms, significant challenges remain for safeguarding biodiversity and ecosystem services in urban areas for the mutual benefit of humans and other species. The majority of future population growth is occurring in places that face some of the most severe challenges to public health and urban biodiversity. In addition, these same urban areas are where systems of formal government and planning tend to be weak (Wilkinson et al. 2013). Together, these patterns suggest a need for increased global support for local governance that takes into account the close relationship between human health and well-being, and the health of local ecosystems.

Effective governance in cities will play a key role in determining the future of biodiversity across the world, not least because cities are rapidly expanding into the world’s biodiversity hotspots. Significant policy changes will need to accompany or even precede effective governance practices, in order to direct future urban growth so that biodiversity and the ecosystems services it provides are safeguarded (Seto et al. 2012; Wilkinson et al. 2013). Ecosystem protection in cities will rely on increasing efforts by park and natural area managers to focus on management outcomes that seek to maximize ecosystem functioning for services, in many places, an abrupt shift from existing or past management goals. Supporting a diversity of governance systems, from official regulations to informal governance systems, for example, local governance of allotment gardens, can provide a multilayered protection system and strengthen support through multiple stakeholders. In addition, developing a blueprint for cross-city comparison of how urban ecosystem services can be assessed (Crossman et al. 2013), safeguarded, and promoted, will be important for understanding which governance mechanisms are generalizable versus those that require more local adaptation.

### Knowledge Gaps

Urban planning, management, and design play a key role in developing cities’ adaptive capacity to the mounting challenges associated with land-use changes (TEEB 2010). However, knowledge is still limited on how local and regional planning and management can serve to reduce mismatches between where ecosystem services are produced, and where they are consumed, while dealing with everyday urban challenges (Haase et al. 2014; McPhearson et al. 2014). Tools and guidelines on how to effectively manage and govern urban ecosystems so that critical services are available to local populations remains an area in need of additional research and practice-based expertise.

The CBO and URBES research outcomes provide significant contributions to the development of new theoretical frameworks and tools for academics, decision-makers, planners, and practitioners alike. Further research and outcomes of these projects have the potential to engage a global audience and to provide scientific knowledge in user-friendly formats for direct dialogue with stakeholders at multiple scales in cities across the world. As the examples with the URBES case-study cities have shown, cities are beginning to take action and increased responsibility for protecting and enhancing the ability of local ecosystems to meet urban resident needs, and the CBO key messages serve as useful guidelines.

### Bridging Knowledge Gaps Vertically and Horizontally

It is increasingly acknowledged that a city and its development are simultaneously dependent upon, and affect the natural landscape, including the ecosystems needed to produce the services consumed in and by urban inhabitants. Following this, it has been argued that the common definition of sustainable development needs to be translated into incorporating a more complex-systems perspective (Seitzinger et al. 2012; Elmqvist et al. 2013). A new definition of urban sustainable development we support is, “a form of development that fosters adaptive and transformative capabilities, and creates opportunities to maintain equitable, long-term prosperity and well-being in complex and interlinked social, economic, and ecological systems” (Elmqvist et al. 2013). Local governments play a vital role in developing the capacity of cities to be adaptive and transformative in the context of their respective needs and challenges. And yet science is needed to provide crucial guidelines and support, providing an understanding of the systems that cities are part of, and developing toolboxes based on insights from other cities—past and present.

The URBES case studies reflect how urbanization is both a challenge and an opportunity to manage ecosystem services intentionally for human well-being. The case studies also demonstrate that cities have a large potential to generate innovations and governance mechanisms that can lead to sustainable development. However, though cities are beginning to take action, an overarching institutional framework for gathering knowledge, furthering the definitions of guidelines, and spurring incentives for action will be crucial. IPBES and Future Earth have emerged to fill this gap to better network scientists globally with practitioners and city leaders for building resilient future cities. Geographic knowledge gaps are, however, still significant since most research on ecosystem services, which is readily available is produced in the US and Europe, followed by Latin America and Asia, with very little from Africa (Elmqvist et al. 2013; Wilkinson et al. 2013).

Connecting local-level decision-makers, planners, and interest organizations with researchers, can thus in a parallel process inform local development and add key insights to the global urban research agenda. The URBES project is one of the first regional research projects to actively use this inclusive approach. These project outcomes can be further underpinned by research-supported capacity-building programmes, enabling practical implementation of scientific knowledge, and guiding the urban development agenda. In a parallel process, networks of cities can support a vertical transfer of knowledge and experiences between the members, encouraging best practices and further increasing the local development capacities.

While the emerging international platforms and networks connect researchers, local decision-makers, planners and experts, the next step to increase the efficiency of policy design and implementation may be to expand these efforts at even more local levels to better connect local authorities and the general public. This is highlighted in the CBO key message 9—*Successful management of biodiversity and ecosystems must be based on multi*-*scale, multi*-*sectoral and multi*-*stakeholder involvement*—which is illustrated by a number of successful examples in *Action and Policy* (Secretariat of the Convention on Biological Diversity 2012) and further discussed in the scientific foundation (Elmqvist et al. 2013). The insights from the URBES project on inclusive governance structures in relatively resource-rich and slow-growing cities (Seitzinger et al. 2012; Westerink et al. 2013; Erixon et al. 2014; Frantzeskaki and Tilie 2014) will need to be adjusted to the growing number of cities around the world with rapidly growing and large populations, often to a large degree consisting of slum dwellers or large migratory populations, with weak ties to official governance structures.

## Conclusion

Protection and sustainable use of ecosystems in cities and in the urban hinterlands are key components of global sustainable development. The CBO Scientific Foundation provides in-depth insights of key elements in sustainability thinking, such as how several development trends can run in parallel, and gives real-world examples of the development trends and challenges faced by cities around the world. Complementing the Foundation, the 10 key messages of the CBO—*Action and Policy* document provide practical guidelines to decision-makers to translate scientific knowledge into actual policies and plans.

Perhaps one of the most important contributions of the URBES and the CBO Projects is the inclusion of regular dialogues with urban policy-makers and planners into the research process, so that knowledge transfer is bidirectional and results can be utilized quickly and efficiently. Yet, further work is required to better understand urbanization patterns and how they will affect URBES in the future, while also creating new tools and improving access to existing tools and best practices for cities to take leadership toward a sustainable, resilient future.

## Footnotes

1 Resilience: the capacity of a system to absorb disturbance and change while still retaining essentially the same identity (Holling 1973; Folke 2010).

2 Sustainable development: to meet the needs of the present without compromising the ability of future generations to meet their own needs (WCED 1987).
